# Engagement, Retention, and Acceptability in a Digital Health Program for Atopic Dermatitis: Prospective Interventional Study

**DOI:** 10.2196/41227

**Published:** 2023-06-14

**Authors:** Sigrídur Lára Gudmundsdóttir, Tommaso Ballarini, María L Ámundadóttir, Judit Mészáros, Jenna Huld Eysteinsdottir, Ragna H Thorleifsdottir, Sigrídur K Hrafnkelsdóttir, Halla Helgadottir, Saemundur Oddsson, Jonathan I Silverberg

**Affiliations:** 1 Sidekick Health Digital Therapeutics Kopavogur Iceland; 2 Department of Health Promotion, Sport and Leisure Studies University of Iceland Reykjavik Iceland; 3 Sidekick Health Digital Therapeutics Berlin Germany; 4 Hudlaknastodin Dermatology Clinic Kopavogur Iceland; 5 Department of Health Sciences, Faculty of Medicine University of Iceland Reykjavik Iceland; 6 School of Medicine and Health Sciences George Washington University Washington, DC United States

**Keywords:** digital therapeutics, DTX, eHealth, engagement, retention, atopic dermatitis, eczema, medication reminder, symptom tracking, patient-reported outcomes, quality of life, dermatology, feasibility, mobile phone

## Abstract

**Background:**

Patients with atopic dermatitis can experience chronic eczema with pruritus, skin pain, sleep problems, anxiety, and other problems that reduce their quality of life (QoL). Current treatments aim to improve these symptoms and reduce inflammation, but poor treatment adherence and disease understanding are key concerns in the long-term management of atopic dermatitis. Digital therapeutics can help with these and support patients toward a healthier lifestyle to improve their overall QoL.

**Objective:**

The aim of the study is to test the feasibility of a digital health program tailored for atopic dermatitis through program engagement, retention, and acceptability.

**Methods:**

Adults with atopic dermatitis were recruited in Iceland for a 6-week digital health program delivered through a smartphone app. Key components of the digital program were disease and trigger education; medication reminders; patient-reported outcomes (PROs) on energy levels, stress levels, and quality of sleep (referred to as QoL PROs); atopic dermatitis symptom PROs; guided meditation; and healthy lifestyle coaching. The primary outcome was program feasibility, as assessed by in-app retention and engagement. User satisfaction was assessed by the mHealth (ie, mobile health) App Usability Questionnaire (MAUQ).

**Results:**

A total of 21 patients were recruited (17 female, mean age 31 years), 20 (95%) completed the program. On average, users were active in the app 6.5 days per week and completed 8.2 missions per day. The education content, medication reminders, and PROs had high user engagement and retention; all users who were exposed to the QoL PROs (n=17) interacted with these, and 20/21 (95%) users were continuously engaged with the education missions, medication missions, and symptom PROs. Continued engagement with the step counter and mind missions among exposed users was lower (17/21 and 13/20 participants, respectively). Medication reminder and education task completion remained high over time (at least 18/20, 90%), but weekly interactions declined. All assigned users completed atopic dermatitis symptom PROs on weeks 1-5 and only one did not do so on week 6; the reported number and total severity of atopic dermatitis symptoms reduced during the program. Regarding the QoL PROs, 16/17 (94%) and 14/17 (82%) users interacted with these at least 3 times in the first and last week of the program, respectively, and all reported improvements over time. User satisfaction was high with a total score of 6.2/7.

**Conclusions:**

We found high overall engagement and retention in a targeted digital health program among patients with atopic dermatitis, as well as high compliance with missions relating to medication reminders, patient education, and PROs. Symptom number and severity were reduced, and QoL PROs improved over time. We conclude that a digital health program is feasible and may provide added benefits for patients with atopic dermatitis, including the tracking and improvement of atopic dermatitis symptoms.

## Introduction

Atopic dermatitis is a chronic, multifactorial, inflammatory skin disease characterized by recurrent eczematous lesions and pruritus [1,2]. The prevalence of atopic dermatitis rose over the last decades, affecting up to 15%-25% of children and 1%-10% of adults worldwide with considerable regional variability [3,4]. Genetic predisposition, environmental factors, defects in skin barrier function, and immune dysregulation are known pathogenic factors; triggers, such as sweating, certain irritants, infections, and stress, can induce eczema and pruritus [2]. The resulting itch-scratch cycle can further damage the skin barrier and hypersensitize affected nerve fibers, some of which also function as pain receptors, and thus, itch is often accompanied by pain [5]. Comorbid health disorders, such as allergies and asthma, sleep problems, and related mental health issues, frequently occur. Hence, atopic dermatitis can significantly impact patients’ quality of life (QoL) and poses a large burden on health care resources [4]. As atopic dermatitis is a chronic disease, it requires treatment over extended periods of time and can be difficult to manage, primarily because of poor treatment adherence—thus, new ways to support patients and improve their self-efficacy are needed [6,7].

One new way to improve treatment adherence and psychological support for chronic diseases is the use of digital therapeutics (DTx) [8], but there are only a limited number of studies about such interventions in atopic dermatitis. One recent randomized controlled study assessed the effect of using medication reminders in the forms of memory buttons with or without a medication tracking app; after 4 weeks, participants in all groups had improved symptom severity, but the effects were more significant in the memory buttons + app group, and in addition, the subjective symptom severity (as measured by the Patient-Oriented Eczema Measure) only improved in this group [9]. Another study evaluated a 12-week-long web-based cognitive behavioral therapy program and found that patients with atopic dermatitis had a significant reduction in the perceived eczema severity, itch, and sleep problems [10]. In addition, a 12-month-long randomized controlled study found that teledermatology consultations were equivalent to in-person visits for improving disease outcomes [11], and a 3-month-long cross-sectional study assessing the engagement with a web-based atopic dermatitis self-management program found that 58% of patients used the program and were most interested in the disease education and exercises [12]. These studies suggest that there is room and potential benefits of digital atopic dermatitis interventions. While self-monitoring of patients in itself can promote self-management by showing patients how their habits and lifestyle affect their recorded data (eg, symptoms) [13], multidisciplinary digital interventions that incorporate additional behavioral change components, psychological and lifestyle support could be the most beneficial for patients [14].

We developed a holistic digital health program to reduce symptom severity and increase the QoL of patients with atopic dermatitis. The program promotes lifestyle changes that focus on habit reversal, treatment adherence, disease education, stress reduction, and sleep improvement. In this brief, 6-week-long study, we evaluated the engagement, retention, and acceptability of this digital health program; clinical efficacy results were published previously [15]. The results of this feasibility study will inform program revisions and determine whether a more extensive study to evaluate clinical efficacy is opportune.

## Methods

### Study Design

A 6-week, single-arm feasibility study was conducted in Iceland between November 2021 and January 2022. Participants’ demographic data were collected at baseline, and they underwent clinical dermatological assessments, performed by a dermatologist, both at baseline and after the 6-week study. Participants were also instructed to install the Sidekick Health app and enroll in the atopic dermatitis program during the baseline visit, and their activities were tracked in the app during the study.

### Participants

Patients with atopic dermatitis who visited the Hudlaeknastodin Dermatology Clinic in Iceland were invited to participate. Patients were screened for inclusion by a study nurse; criteria were (1) mild to severe atopic dermatitis, (2) ≥18 years of age, (3) understands verbal and written Icelandic, (4) capacity to consent, and (5) owns and knows how to operate a smartphone. Exclusion criteria were (1) recent (within 4 weeks) or current phototherapy or oral treatment for atopic dermatitis; (2) other inflammatory skin diseases (ie, nummular dermatitis, seborrheic dermatitis, contact dermatitis, and psoriasis); (3) pregnancy; and (4) any biological treatment for atopic dermatitis. Participants’ demographic and background information were collected at baseline at the clinic.

### Digital Program

A digital health program for patients with atopic dermatitis was developed by Sidekick Health, a digital therapeutics company [14,15]. The program was delivered through a mobile phone app, designed to engage patients with atopic dermatitis in lifestyle optimization and increase their treatment adherence.

The program was composed of education about the disease and how lifestyle affects disease progression; patient-reported outcomes (PROs) on symptoms and quality of sleep, stress, and energy levels; medication reminders; and advice and support on healthy lifestyle provided by a coach. To achieve behavioral modification, participants received these contents as missions throughout the program, which were designed to help them adjust their behavior according to the challenges they face when dealing with atopic dermatitis. Missions were defined as all assignments available in the app; participants were assigned on average 5 missions per day from the above categories that they were asked to complete. In addition, users could complete “open missions” such as guided meditation and logging steps (with a step counter), which were not assigned by the program specifically that day.

1. *Education* was composed of videos and content cards relating to atopic dermatitis, skin care, symptoms, triggers, comorbidities, stress, sleep, and mindset.
2. *PROs on atopic dermatitis symptoms* were assigned once a week on a specific day (thus, users were not assigned if they did not visit the app on the given day). Users were first asked to select which of the listed symptoms they had experienced in the past week; the options were itch, bleeding skin, cracked skin, flaking skin, dry or rough skin, weeping or oozing clear fluid from eczema, disturbed sleep, or none of these. Based on the response from the first question, users were then asked to rate the severity of their symptoms ranging from 1 (mild) to 9 (severe). The total score per participant was calculated by adding up the ratings from all questions. If a participant responded with “none of these,” then the total score for that week was zero.
3. *PROs on quality of sleep, stress, and energy levels* were collected 3 times per week from week 2 onward, where participants rated themselves on a 10-point sliding visual analog scale.
4. *Medication reminders* were set up by the participants with customized timings and frequency. Participants then received the reminder as a mission each time, which was completed when the user pressed “done.”

### Outcomes Definition and Analysis

Program engagement was assessed by determining program usage patterns: how often participants engage with the content, how many tasks were performed, and what types of tasks (eg, medication reminder and symptom PROs) were performed. Dedicated users were defined as participants who were active for at least 5 days (or more) every week. The engagement and retention for key app features—education, medication, PROs, step count, and mind exercises—are reported as the number of users calculated in the following four stages: (1) *exposed*: the number of users passively exposed to the feature, no action was required; (2) *interacted*: those who interacted during the first week of exposure; (3) *activated*: those who used the feature again after activation; and (4) *retained*: those who used the feature in the last week.

Acceptability of the program was assessed through the mHealth App Usability Questionnaire (MAUQ) for standalone apps, which was administered to participants after the study [16]. The MAUQ is an 18-item questionnaire that measures 3 factors: ease of use (MAUQ_E, 5 items), interface and satisfaction (MAUQ_I, 7 items), and usefulness (MAUQ_U, 6 items). Responses to the statements on the MAUQ ranged from 1 (strongly disagree) to 7 (strongly agree). To obtain 3 subscores (MAUQ_E, MAUQ_I, and MAUQ_U), as well as an overall score (MAUQ total), the average of the corresponding items was computed for each individual. Median scores and IQRs were calculated for the group.

Descriptive statistics were computed as mean and SD or median and IQR for continuous variables and numbers and percentages for categorical ones. The longitudinal changes in QoL and symptom PROs were tested by comparing the first and the last available measures using paired-sample *t* tests (α=.05). R (version 4.1.2; R Foundation for Statistical Computing) was used for all analyses.

### Ethics Approval

The study protocol was approved by the Icelandic National Bioethics Committee (institutional review board registration number VSNb2021090028/03.01). All participants were given detailed explanation of study procedures and signed informed consent (in Icelandic) prior to enrollment. All study data were anonymized, and the information collected in the app was encrypted and handled in compliance with General Data Protection Regulation.

## Results

### Demographics and Program Engagement

A total of 21 adult patients were recruited in the study, 20 (95%) of whom completed the program. Information about participants and their average app usage is presented in Table 1; a more detailed demographic description was described elsewhere [1]. Participants were highly engaged with the app as they used it almost every day (6.5 days per week on average) and completed an average of 8.2 missions a day (Table 1).

**Figure 1:**
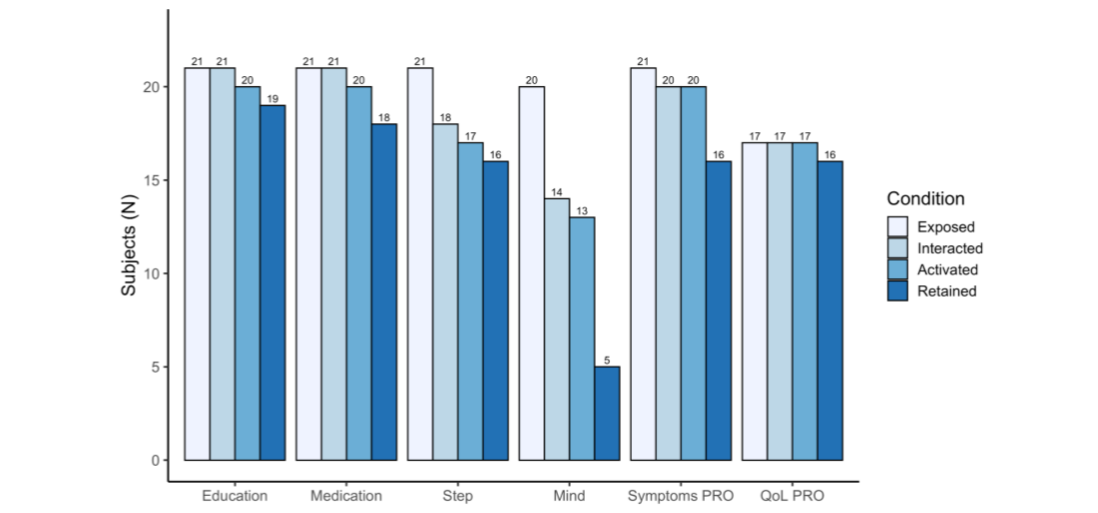
Adoption of key app features. Exposed: number of users passively exposed to the feature, no action was required; interacted: number of users who interacted during the first week of exposure; activated: number of users who used the feature again after activation; and retained: number of users who used the feature in the last week. PRO: patient-reported outcome; QoL: quality of life.

Breakdown of the adoption of key app features (medication reminder, education content, step counter, mind missions, symptom PROs, and QoL PROs) showed that participants used the medication reminders, QoL PROs, and disease education most consistently throughout the program (Figure 1). These features had the highest user retention until week 6, and the QoL PROs had constant continued engagement and retention (n=17). Atopic dermatitis symptom PROs also had high compliance, as almost all users completed these when assigned (20/21, 95%) with only 4 people not complying at week 6. The step counter had moderate interactions, as most users (17/18, 94%) continuously recorded their steps in the app with only 1 participant who stopped using this feature in week 6, and the mind missions had the lowest number of interactions (14/20, 70%) and retention (5/13, 38%). It should be noted that the step count and the mind missions were open tasks, meaning that they were not assigned as daily tasks but the users could proactively complete them.

Table 2 presents weekly user engagement with all missions and the completion rates of medication reminder and education missions when assigned. On average, users interacted more with the app in the first 2 weeks (10 times per day) compared with the last 4 weeks (7-8.9 times per day), but user interaction remained high throughout the program and only 1 user dropped out after the first week.

**Figure 2:**
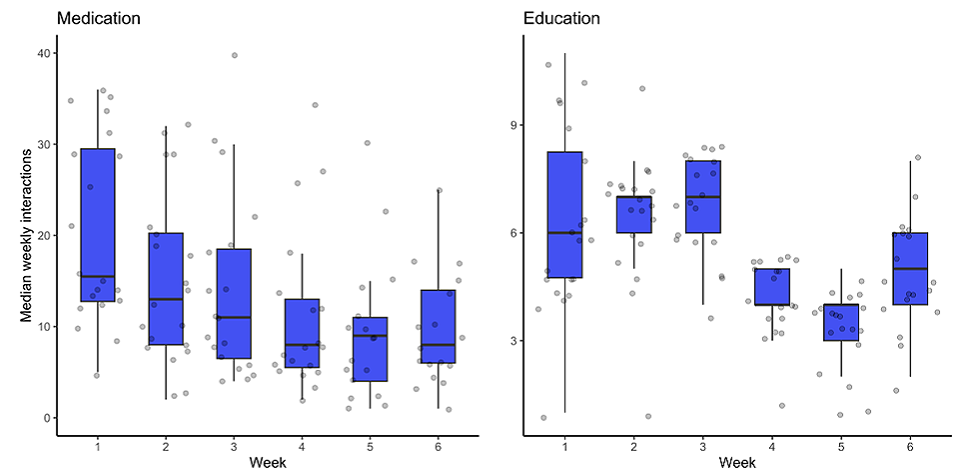
Mean weekly interactions with medication reminders and education content.

Medication reminders and education content missions both had high weekly compliance rates; all assigned users (n=20) completed these tasks in the first 2 weeks, and completion remained over 90% for all subsequent weeks (Table 2). However, on average, users interacted less frequently with the medication tasks over time (median weekly interaction was 15.5 days on week 1 vs 8.5 days on week 6; Figure 2). The education content had a median weekly interaction of 6.0 on week 1 and 5.0 on week 6, and participants remained highly engaged with this type of content in the first half of the program and less so in the second half (Figure 2).

**Table 1:** Overview of participants and program engagement.

Variable	Value
Age (years), median (IQR)	31 (26-37)
Female, n (%)	17 (81)
Average active days per week (0-7), median (IQR)	6.5 (5.5-6.8)
Average total active days (0-42), median (IQR)	39.0 (33.0-41.0)
Average daily mission interactions, median (IQR)	8.2 (7.1-9.7)
Dedicated users, n (%)	12 (57)

**Table 2:** Weekly breakdown of program engagement and completion of medication reminders and education content.

Week number	Daily mission interactions (N=21), mean (SD)	Medication reminders completion^a^	Education content completion^b^
		Users, n (%)	Users, n (%)
1	10.3 (5.1)	20 (100)	20 (100)
2	10.2 (4.1)^c^	20 (100)	20 (100)
3	8.6 (3.4)^c^	19 (95)	19 (95)
4	8.9 (3.2)^c^	19 (95)	19 (95)
5	7.0 (2.9)^d^	17 (94)	19 (100)
6	8.6 (3.4)^c^	18 (90)	19 (95)

^a^N=20 assigned users, except on week 5: N=18 assigned users.

^b^N=20 assigned users, except on week 5: N=19 assigned users.

^c^Data available for N=20.

^d^Data available for N=19.

### In-App PROs

**Figure 3:**
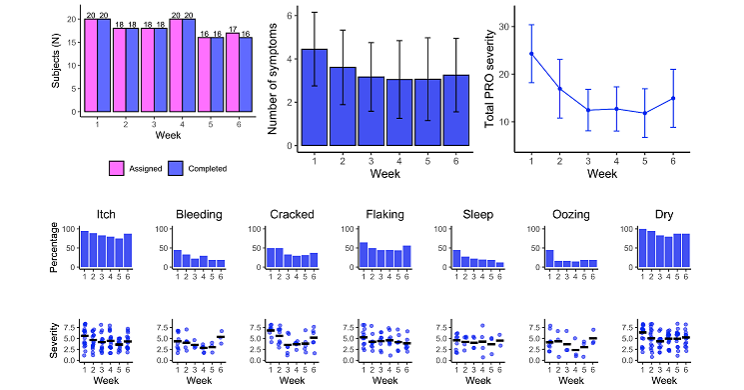
Completion of atopic dermatitis symptom PROs, and the number and severity of reported symptoms. PRO: patient-reported outcome.

We analyzed the weekly engagement with atopic dermatitis symptom PRO surveys as well as the reported number and severity of symptoms (Figure 3) and found that all assigned users completed the symptom surveys each week with only 1 missing user in the last week. The number of assigned users differed between weeks because these missions were not assigned if a participant already had many other types of missions to complete that week. This was done to avoid overwhelming participants with different types of content. The average number of symptoms as well as the average symptom severity decreased from week 1 to 3 and stayed stable until week 6. The most commonly reported symptoms were dry skin and itch as nearly all participants reported these in the first week (20/20, 100% and 19/20, 95%, respectively), and their frequency remained high throughout the study relative to other symptoms. Itch and dry, flaking, or cracked skin were the most severely rated in the first 2 weeks, while cracked skin, bleeding and oozing from the lesions, and eczema-related sleep problems were rarely reported and at low severity in the last 4 weeks.

**Figure 4:**
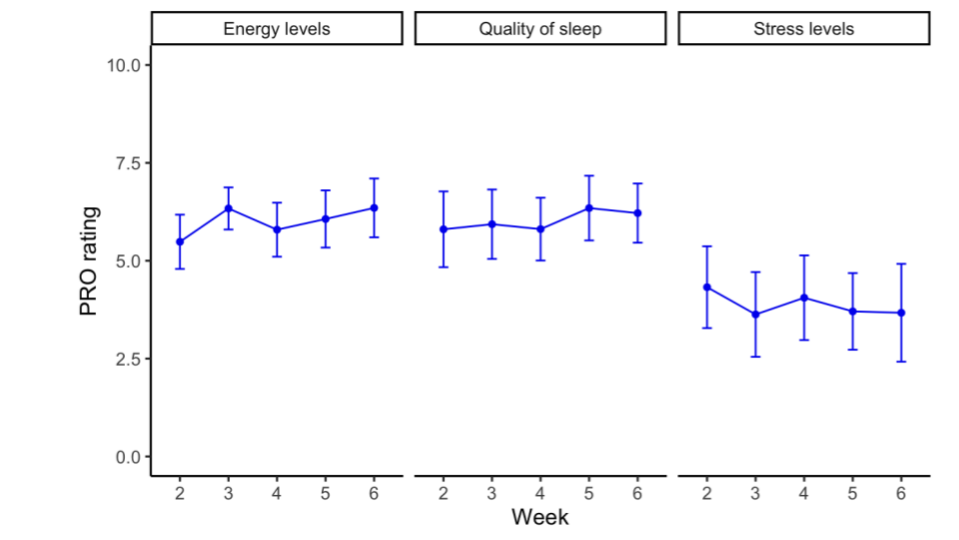
Changes in patient-reported sleep, stress, and energy levels over time. PRO: patient-reported outcome.

PROs on energy levels, sleep quality, and stress levels showed that of the 17 assigned users, 16 (94%) reported these at least 3 times in week 1 and 12 (70%) continued to do so in week 5 (Table 3), demonstrating high user compliance. The average reported energy levels increased significantly by 15% from week 1 to 6 (*P*=.006), and there was a trend toward overall improved sleep quality (7%; *P*=.39) and reduced stress levels (13%; *P*=.19; Figure 4).

**Table 3:** Engagement with QoL^a^ patient-reported outcomes.

Week number	QoL (energy, sleep, and stress),^b^ n (%)	
	≥1 interaction per week	≥3 interactions per week
1	—^c^	—
2	17 (100)	16 (94)
3	16 (94)	13 (76)
4	17 (100)	15 (88)
5	17 (100)	12 (70)
6	16 (94)	14 (82)

^a^QoL: quality of life.

^b^Expressed as percentage of assigned users (N=17).

^c^Not available.

### User Satisfaction

User satisfaction was high, as measured by the MAUQ. The median total MAUQ score was 6.2 out of 7 (N=20; IQR 5.4-6.5), and the median scores for ease of use, interface satisfaction, and usefulness were 5.9 (IQR 5.6-6.4), 6.4 (IQR 5.4-6.9), and 6.3 (IQR 5.3-6.7), respectively.

## Discussion

### Principal Results

We found high overall program engagement and user retention in this 6-week-long study, suggesting that a holistic digital health program is feasible for patients with atopic dermatitis. We also analyzed user activity flow over time for key app features and created 4 categories: those users who were passively exposed to a specific feature (exposed), those who then actively interacted with it (interacted), those who continued using the feature (activated), and those who used the feature in the last week (retained). This analysis showed that users complied with the missions in most categories when these were assigned. Particularly, PROs, medication reminders, and educational materials had high compliance, suggesting that participants were interested in self-assessment, learning more about their disease, and acquiring help for managing medication schedules. These results were consistent with another similar 3-month long study where participants interacted the most with the education parts about atopic dermatitis and its treatments, in particular [1]. It remains to be seen in longer studies whether patients’ engagement and retention in these domains remain high or decrease over time. Maintaining interest and engagement with such features over longer periods of time (ie, months and potentially years), as required for chronic diseases, will be a challenge for DTx [17,18].

Detailed analysis of engagement with medication reminders and educational materials corroborated the above findings regarding high user compliance. We found that, while medication mission completion remained stable over time, the average weekly interactions with these tasks gradually decreased. This could be interpreted as people setting fewer medication reminders or deleting them over time as their symptoms improved, while they continued completing those that they kept. Indeed, symptom PROs showed that the average reported number and severity of atopic dermatitis symptoms reduced over time.

Similar to medication reminders, we also found an initial high interaction with the education content (weeks 1-3) that decreased after week 4. This can be explained by users exhibiting an initial exploratory behavior—while they actively seek out education content—followed by an “as-necessary” phase when users only complete the missions that were assigned to them. Decreased user engagement with DTx programs is expected and is well documented [17,18], but—especially in the case of educational materials—this can be due to users reducing their app usage when they are content with the amount of knowledge they have gained. Taken together, the above findings suggest that the app successfully engages users, which may help them adapt behaviors leading to improved disease symptoms and better overall health.

Another important finding was the feasibility of continuous patient monitoring, as demonstrated by the high compliance with both the symptom and QoL PRO missions. However, there was no control over what time of the day participants rated their sleep quality, energy, or stress levels, which may increase the day-to-day variability of these measurements—future studies will need to further validate the clinical accuracy and reliability of in-app PROs.

Several previous studies have assessed the efficacy of multidisciplinary interventions among adult patients, or pediatric patients and their parents [14]. These interventions were designed to address multiple aspects of atopic dermatitis management, such as disease and trigger education, treatment adherence, and psychological tools for managing itch and pain. Two studies reported decreased symptom severity among adults [19,20], and several studies found improved symptoms, as well as increased QoL in parents and pediatric patients [14]. Thus, adult patients with atopic dermatitis can benefit from a holistic treatment program, and digital interventions provide new ways to deliver these. However, a review of 98 eczema management apps found that none of the apps met the full criteria for the education and health-tracking functionalities provided based on international eczema management guidelines. For example, most apps targeting adult patients lacked educational content about treatment side effects, pharmacological or nonpharmacological information from valid sources, or the ability to track disease status or trigger factors [21]. The digital health program evaluated in this study included functionalities for most (though not all) assessment criteria points from the above review.

### Limitations

This study was limited by a small sample size and the relatively homogeneous sample; larger, multicenter trials would need to assess the generalizability of these results among a more diverse group of patients. The statistical comparison of QoL and symptom PROs from first to last measurements did not include a control group and should hence be interpreted with caution. In addition, while here we report high medication adherence results, it should be noted that completion of a medication reminder does not guarantee treatment completion (that the patient actually took the medication or applied the topical treatment). Another limitation of the study was that not all mission categories analyzed here were assigned for users (namely, step counter and meditation); therefore, it is difficult to compare the engagement and retention with other assigned mission categories.

### Conclusions

To our knowledge, this is the first study to assess the feasibility of a holistic digital health program for patients with atopic dermatitis. The high continued program engagement and retention, compliance with medication reminders, education content, symptom, and QoL PROs, and the high user satisfaction provide strong evidence for the feasibility of an atopic dermatitis–specific digital solution for patients. In addition, the decrease in symptom frequency and severity, as well as improved ratings of sleep quality, energy, and stress levels recorded in the app agree with previously reported results of clinical assessments of these patients. These results can inform the design of future randomized controlled trials to further validate the efficacy of a targeted DTx program in atopic dermatitis.

## Abbreviations

DTx: digital therapeutics
MAUQ: mHealth App Usability Questionnaire
PRO: patient-reported outcome
QoL: quality of life

## Data Availability

The anonymized data sets generated and analyzed in this study are available from the corresponding author upon request.
